# Estimation of genetic parameters for fertility traits in Chinese Holstein of south China

**DOI:** 10.3389/fgene.2023.1288375

**Published:** 2024-01-03

**Authors:** Kai Zhu, Tuowu Li, Dengying Liu, Shiyi Wang, Sihu Wang, Qishan Wang, Yuchun Pan, Linsen Zan, Peipei Ma

**Affiliations:** ^1^ College of Animal Science and Technology, Northwest A&F University, Yangling, Shaanxi, China; ^2^ Bright Dairy & Food Co., Ltd., Shanghai, China; ^3^ Shanghai Collaborative Innovation Center of Agri-Seeds, School of Agriculture and Biology, Shanghai Jiao Tong University, Shanghai, China; ^4^ Department of Animal Science, College of Animal Sciences, Zhejiang University, Hangzhou, China; ^5^ National Beef Cattle Improvement Center, Northwest A&F University, Yangling, Shaanxi, China

**Keywords:** Chinese Holstein, South China, fertility traits, genetic parameters, heritability

## Abstract

**Introduction:** Chinese Holstein in South China suffer heat stress for a long period, which leads to evolutionary differences from Chinese Holstein in North China. The aim of this study was to estimate the genetic parameters of fertility traits for Chinese Holstein in South China.

**Methods:** A total of 167,840 Chinese Holstein heifers and cows from Guangming Animal Husbandry Co., LTD farms were used in this study. The fertility traits analyzed were calving interval (CI), days open (DO), age of first service (AFS), age of first calving (AFC), calving to first insemination (CTFS), first insemination to conception (FSTC), gestation length (GL), non-return rate to 56 days (NRR), and number of services (NS).

**Results:** The descriptive statistics revealed that the same trait in heifers performed better than in cows, which was consistent with the other studies. The heritabilities of fertility traits in this study ranged from close to 0 (for NS of cows) to 0.2474 (for AFC of heifers). The genetic correlation of NRR between heifers and cows was 0.9993, which indicates that the NRR for heifers and cows could be treated as one trait in this population.

**Conclusion:** The heritabilities of fertility traits in Chinese Holstein in south China were quite different from the heritabilities of fertility traits in North China. NRR56, NS, AFC, and CI were suggested to be included into the selection index to improve fertility performance of Chinsese Holstein of south China. The results of this study could provide genetic parameters for the animal breeding program of Chinese Holstein in the south of China.

## 1 Introduction

Reproduction traits are the key to postpartum lactation and herd expansion, and directly affect the productive life of dairy cows. Therefore, the reproductive traits should be considered as among the most economically important traits. Most studies showed the genetic correlation between reproductive traits and milk yield traits was negative (Pryce et al., 2004; Walsh et al., 2011). The high-intensity selection of milk yield traits leads to the gradual decline of the reproductive traits of dairy cows. Therefore, it is crucial to improve the selection of reproductive traits in dairy cows. Investigating the genetic parameters is the basic procedure for the selection of reproductive traits. The genetic parameters are population-specific, therefore, it is necessary to estimate the genetic parameters in different populations. The genetic parameters of reproductive traits in Chinese Holstein cattle have been estimated using the Beijing population (Guo et al., 2014; Liu et al., 2017), the Ningxia population (Chen et al., 2021), and so on.

Guo et al. (Guo et al., 2014) evaluated the genetic parameters of five reproductive traits of dairy cows from Sanyuan Lvhe Dairy Cattle Center, Beijing, China. The traits were age at first service (AFS), number of services (NS), days from calving to first service (CTFS), days open (DO), and calving interval (CI). The range of heritabilities for these traits was from 0.034 to 0.100. The heifer and cow records were mixed together for the estimation of genetic parameters in the study of Guo et al. (Guo et al., 2014). Nevertheless, the physiology of cows is significantly different from that of heifers, so the genetic parameters should be estimated separately. Furthermore, Liu et al. (Liu et al., 2017) re-estimated the genetic parameters of interval from calving to first insemination, interval from first to last insemination, days open, conception rate at first insemination, number of inseminations per conception, and non-return rates within 56 days after first insemination for heifers and cows separately using a larger population in 2017 (Liu et al., 2017). The study of Liu et al. (Liu et al., 2017) was also based on the population of the Sanyuan Lvhe Dairy Cattle Center in Beijing.

In southern China, the useful life of dairy cows is shorter than in northern China due to heat stress, and one of the important reasons for culling cows is the passive culling of reproductive traits (Liu et al., 2021). Previous studies have also indicated that reproductive traits in dairy cattle are strongly negatively affected by tropical climate conditions (heat stress). Multiple parameters including large follicles, corpora lutea, ovulation rate, fertilized ova, and transferable embryos were influenced at a temperature–humidity index (THI) of 72 but significantly affected after a THI of 77 (*p* < 0.05) (Ratchamak et al., 2021).

In our previous study, we found that due to the wide geographical area in China, there was an apparent genetic and environmental interaction between the northern and southern populations for the most economic traits. Therefore, it is necessary to estimate the genetic parameters of reproductive traits in Southern Chinese Holstein cattle populations.

## 2 Materials and methods

The population used in this study was collected from Guangming Animal Husbandry Co., LTD. The farms were located in South China including Shanghai City, Jiangsu province and Zhejiang province. The data were edited to remove the feasible errors in the records.

Firstly, we merged the reproductive trait records uploaded from 32 farms from 2007 to 2020, and removed duplicate records based on the cow identification number and parity, finally obtaining a sum of 141,933 reproductive records. Secondly, due to the different formats of tables for recording reproductive traits data in each farm, the types of reproductive traits recorded were also different. Therefore, the total table was split into multiple sub-tables for recording different reproductive traits. After removing duplicate records, we got 56,624 records for NS, 91,301 records for CI, 83,727 records for gestation length (GL), and 178,121 records for age of first calving (AFC, part of DHI data). Other reproductive traits can be calculated from the records in the sub-table, such as CTFS, which can be obtained by subtracting the calving time from the first insemination time in the NS file. However, due to the fact that our pedigree file matches milk production traits and does not fully match reproductive traits, we were required to remove individuals from reproductive trait records not included in the pedigree file based on existing pedigree information. At the same time, we also found some mistakes in the record file including the same cattle number corresponding to multiple cows, which was an error in the birth record of cows with abnormal birth times as the cow identification number was consistent while other information records were inconsistent. The records larger than four times the standard deviation were also removed to control the outliers in the dataset. After eliminating the above mistakes, our reproductive trait records included 41,884 records for NS, 49,634 records for CI, 74,180 records for GL, 168,170 records for AFC, 8,501 records for AFS, 41,821 records for first insemination to conception (FSTC), and 49,634 records for CTFS. The non-return rate to 56 days (NRR) was coded as 0 or 1. The animal return to estrus within 56 days after calving was 0, otherwise it was 1. The pedigree was also provided by Guangming Animal Husbandry Co., LTD. The individuals with this phenotype were traced back to as many generations as possible. Finally, the best linear unbiased prediction (BLUP) model was used in this study. DMU software (Madsen et al., 2014) was used to implement the restricted maximum likelihood (REML).

$$y=Xb+\sum Z_{i}u_{i}+e$$

In which y is the phenotype vector; b is the fixed effects (Table 1 for each trait); $u_{i}$ is the random effects, including a , **HY,** or SS (Table 1 for each trait) (Madsen et al., 2014); $a∼N\left(0,A\sigma_{a}^{2}\right)$ is the additive genetic effects (i.e., estimated breeding values for each individual); A is the additive genetic relationship matrix; and $\sigma_{a}^{2}$ is the additive genetic variance. **HY**∼ $N\left(0,I\sigma_{HY}^{2}\right)$ is the herd-year effect. SS **∼** $N\left(0,A_{SS}\sigma_{ss}^{2}\right)$ is the service sire effect, $\sigma_{ss}^{2}$ is the service sire variance, and $A_{SS}$ is the additive genetic relationship matrix only for service sire X **.** $Z_{i}$ is the design matrix for b and $u_{i}$ **.** e is the residual effect with the distribution $N\left(0,I\sigma_{e}^{2}\right)$ , and the $\sigma_{e}^{2}$ is the residual variance.

**TABLE 1:** Fixed and random effects included in the genetic model for genetic parameter estimation for heifer (○)and cow (●) traits.

Trait	Fixed effect							Random effect		
	RYM	Mf	MfX	AcMcX	ApMf	ApMp	ApMpX	HY	SS	a
AFS	○							○		○
AFC	○							○		○
NRR	○●	○			●			○●	○●	○●
NS	○●	○			●			○●		○●
FSTC	○●	○			●			○●		○●
GL	○●		○				●	○●	○●	○●
DO	○●			○●				○●		○●
CTFS	●					●		●		●
CI	●					●		●		●

a : random animal effect; AcMcX: age at current calving by month of current calving by sex of calf by parity; AFC: age at first calving; AFS: age at first insemination; ApMf: age at previous calving by month of first insemination by parity; ApMp: age at previous calving by month of previous calving by parity; ApMpX: ApMp by sex of calf; CI: calving interval; CTFS: calving to first insemination; DO: days open; FSTC: first insemination to conception; GL: gestation length; HY: herd by year of birth; MF: month of first insemination; MfX: month of first insemination by sex of calf; NRR: non-return rate to 56 days; NS: number of services; RYM: region by year of birth by month of birth; SS: service sire.

The two-trait model was used to estimate the genetic correlations of the heifer and cow populations.

$$\left[\begin{array}{c} y_{1}\\ y_{2} \end{array}\right]=\left[\begin{array}{cc} X_{1} & 0\\ 0 & X_{2} \end{array}\right]\left[\begin{array}{c} b_{1}\\ b_{2} \end{array}\right]+\sum \left[\begin{array}{cc} Z_{1i} & 0\\ 0 & Z_{2i} \end{array}\right]\left[\begin{array}{c} u_{1i}\\ u_{2i} \end{array}\right]+\left[\begin{array}{c} e_{1}\\ e_{2} \end{array}\right]$$

In the above formula, $y_{1}$ is the vector of heifer phenotype, $y_{2}$ is the vector of cow phenotype. $b_{1}$ , $b_{2}$ is the fixed effects (Table 2) for heifer traits and cow traits, respectively. $u_{1i}$ **,** $u_{2i}$ are the random effects (Table 2 for each trait), and $a_{1}$ and $a_{2}$ are the estimated breeding values for heifer traits and cow traits, respectively. $\left[\begin{array}{c} a_{1}\\ a_{2} \end{array}\right]$ follows the distribution $N\left(0,A⊗\left[\begin{array}{cc} \sigma_{a_{1}}^{2} & \sigma_{a_{1}}\sigma_{a_{2}}\\ \sigma_{a_{1}}\sigma_{a_{2}} & \sigma_{a_{2}}^{2} \end{array}\right]\right)$ . The distribution for service sire effects was similar to the estimated breeding value. $e_{1}$ , $e_{2}$ is the residual errors with the distribution $N\left(0,I⊗\left[\begin{array}{cc} \sigma_{e_{1}}^{2} & \sigma_{e_{1}}\sigma_{e_{2}}\\ \sigma_{e_{1}}\sigma_{e_{2}} & \sigma_{e_{2}}^{2} \end{array}\right]\right)$ . The distribution for herd-year effects was similar to the residual errors. $X_{1}$ , $X_{2}$ , $Z_{1i}$ **,** and $Z_{2i}$ are the design matrices for $b_{1}$ , $b_{2}$ , $a_{1}$ and $a_{2}$ , respectively.

**TABLE 2:** The descriptive statistics for reproductive traits in heifers and cows.

Trait	N	Mean	SD	Minimum	Maximum
CI (cow)	43,292	429.19	100.05	265	838
DO (cow)	26,766	156.75	100.21	10	560
AFS (heifer)	6,215	467.48	45.83	284	897
AFC (heifer)	167,840	781.87	77.05	570	1,096
CTFS (cow)	10,981	80.22	30.78	3	225
FSTC (cow)	11,032	61.83	81.86	0	399
FSTC (heifer)	6,065	20.31	36.89	0	188
FSTC (all)	17,097	47.10	72.11	0	399
GL (cow)	18,171	276.68	8.23	230	322
GL (heifer)	2,866	278.42	6.82	248	308
GL (all)	21,037	276.92	8.07	230	322
NRR (cow)	10,587	0.4576	0.4982	0	1
NRR (heifer)	6,218	0.2969	0.4569	0	1
NRR (all)	16,805	0.3982	0.4895	0	1
NS (cow)	11,052	2.25	1.52	1	9
NS (heifer)	6,188	1.58	0.94	1	6
NS (all)	17,240	2.01	1.38	1	9

CI, calving interval; DO, days open; AFS, age of first service; AFC, age of first calving; CTFS, calving to first insemination; FSTC, first insemination to conception; GL, gestation length; NRR, non-return rate to 56 days; NS: number of services.

## 3 Results

The descriptive statistics are shown in Table 2. The pedigree data contained 529,011 individuals, which indicated that most heifer traits performed better than cows. The NS in heifers was 1.58 on average while it was 2.01 for cows. The larger NS meant that more services were needed for conception. The NRR was 0.2969 on average for heifers and 0.4576 for cows, which suggested that cows were difficult to induce to conceive. The FSTC was 20.31 and 61.83 for heifer and cow respectively, which indicated that cows would need more days from first service to conception.

The variance components and heritability of each trait calculated using the animal model BLUP are shown in Table 3. It can be seen from the results that the heritability of each reproductive trait of Holstein cattle in southern China is relatively low. The lowest one was close to 0, which was from the NS of cows. The highest one was 0.2474, which was from the AFC of heifers. AFS and AFC were only recorded for heifers while CI and DO were only recorded for cows. The heritabilities were 0.0608 and 0.2474 for AFS and AFC, and the heritabilities for CI and DO were 0.0118 and 0.0168. Generally, it was difficult to find whether the heritability for heifers was higher or whether that for cows was higher. For example, the heritability for GL in heifers was 0.0494 which was lower than for cows (0.1250). However, the heritability of NS for heifers was 0.0549, which was higher than for cows.

**TABLE 3:** Variance component and heritability of reproductive traits in heifers and cows.

Trait	${\hat{\sigma }}_{g}^{2}$	${\hat{\sigma }}_{hy}^{2}$	${\hat{\sigma }}_{ss}^{2}$	${\hat{\sigma }}_{e}^{2}$	${\hat{h}}_{g}^{2}$
CI (cow)	109.14 (25.57)	446.55 (58.57)		8,690.96 (74.03)	0.0118 (0.003)
DO (cow)	155.28 (50.66)	208.06 (45.62)		8,894.42 (89.22)	0.0168 (0.005)
AFS (heifer)	215.09 (56.18)	2,149.04 (586.76)		1,172.98 (51.67)	0.0608 (0.02)
AFC (heifer)	1,137.22 (33.59)	1,300.42 (52.07)		3,462.47 (26.98)	0.1927 (0.0056)
CTFS (cow)	0.71 (5.03)	30.06 (8.46)		860.05 (12.83)	0.0008 (0.006)
FSTC (cow)	43.13 (44.46)	179.42 (55.51)		6,130.55 (97.85)	0.0068 (0.007)
FSTC (heifer)	115.14 (35.40)	245.44 (80.99)		1,105.28 (36.63)	0.0785 (0.024)
GL (cow)	8.52 (0.97)	1.19 (0.36)	1.00 (0.24)	57.46 (0.95)	0.1250 (0.014)
GL (heifer)	2.31 (1.39)	4.59 (2.06)	0.66 (0.40)	39.24 (1.64)	0.0494 (0.03)
NRR (cow)	0.34E-03 (0.0012)	0.94E-02 (0.0028)	0.19E-01 (0.0038)	0.22 (0.0035)	0.0014 (0.0049)
NRR (heifer)	0.40E-04 (0.002)	0.23E-01 (0.008)	0.41E-01 (0.0065)	0.17 (0.0037)	0.0002 (0.0085)
NS (cow)	0.76E-07 (0.0113)	0.12 (0.0316)		2.14 (0.033)	0.0000 (0.005)
NS (heifer)	0.50E-01 (0.0186)	0.17 (0.054)		0.74 (0.021)	0.0549 (0.019)

CI, calving interval; DO, days open; AFS, age of first service; AFC, age of first calving; CTFS, calving to first insemination; FSTC, first insemination to conception; GL, gestation length; NRR, non-return rate to 56 days; NS, number of services.

A two-trait model was used to estimate the genetic correlation for each trait between heifers and cows. Unfortunately, only the two-trait model for NRR converged. The data for heritability and genetic correlation of NRR are shown in Table 4. Heritability using the two-trait model was improved for both heifers (0.0031 vs. 0.0002) and cows (0.0022 vs. 0.0014). The genetic correlation was 0.9993 while the phenotypic correlation was 0.1475.

**TABLE 4:** The heritability (diagonal) and the genetic correlation (lower triangle) of NRR using a two-trait model.

	NRR (heifer)	NRR (cow)
NRR (heifer)	0.003 (0.10)	
NRR (cow)	0.9993 (3.379)	0.0022 (0.005)

NRR: non-return rate to 56 days.

## 4 Discussion

This study investigated the genetic parameters of heifers and cows in southern China. Animal models were used and genetic correlations between heifer and cow populations were estimated. The heritabilities of reproductive traits in this study were similar to the other Holstein populations. The genetic correlation of NRR between heifers and cows was 0.9993, which indicated that this trait in heifers and cows could be treated as one trait.

Compared with the results from the other studies, the performance of all the traits in the present study was in the middle (Sun et al., 2010; Guo et al., 2014; Buaban et al., 2016; Liu et al., 2017; Haile and Makasha, 2018; Muuttoranta et al., 2019; Alves et al., 2020; Sharko et al., 2022). The average of most traits in this study was superior to that of the Chinese Holstein populations in north China, Thailand, Russia, and Ethiopia which were reported from 2011 to 2022 (Buaban et al., 2016; Liu et al., 2017; Haile and Makasha, 2018; Sharko et al., 2022). The FSTC for heifers and cows was 32.1 and 79.5 in north China respectively, and the FSTC for cows from Thailand and Russia was 55.34 and 41.685 respectively. In this study, the average FSTC for heifers and cows was 20.31 and 61.83, respectively. The difference was more than 10 days compared with cows from north China. However, the FSTC of cows was longer than cows from Thailand and Russia. The DO was 156.75 in this study while it was 161.6 in the population in north China, 156.3 in Thailand, and 140.18 in Russia. The NS for heifers and cows in this study was 1.58 and 2.25, respectively, while they were 1.742 and 2.777 in the north China Holstein population. The CTFS in this study compared to the other study was 80.22, as opposed to 82.1. Both were shorter than that in the Nordic Holstein population (Muuttoranta et al., 2019), the Netherlands population (de Haer et al., 2013), and the American population (Norman et al., 2009). We found that the minimum CTFS was 3 and the maximum was 225 from the descriptive statistics, which showed unreasonable records. Therefore, it is hard to compare phenotypic levels in different countries since the data selection and editing were diverse in these studies. Furthermore, the results did not indicate that dairy farms in China paid more attention to management compared with the other countries. The comparison between heifers and cows was consistent with other studies (Guo et al., 2014; Liu et al., 2017; Muuttoranta et al., 2019). The traits of heifers were superior to those of cows. The reasons could be that the physiological effects of cows altered after calving and the high yield resulted in a negative energy balance (Wiltbank et al., 2006).

The results of this study demonstrated that the heritability of various reproductive traits of Holstein cattle in southern China was smaller than 0.1 except for the AFC of heifers and GL of cows, which was consistent with the previous studies (Jamrozik et al., 2005; Veerkamp and Beerda, 2007; Liu et al., 2008). According to the results from a review study (Pryce and Veerkamp, 2001), the heritabilities of NRR, NS (cow), CTFS, and FSTC were smaller than the average, and/or also smaller than the results from northern China. The heritabilities of NS(cow), CTFS and FSTC were also smaller than the results from Thailand and Russia (Buaban et al., 2016; Liu et al., 2017; Sharko et al., 2022). The reasons might be due to irregular individual management and the greater influence of environmental factors. In southern China, the environmental temperature is too high for raising Holstein cattle, which usually leads to heat stress in summer for most individuals, resulting in a high incidence of individual mastitis and other diseases, and also leads to a short service life (Liu et al., 2021), which might indirectly affect the heritability of reproductive traits. We investigated four traits for both heifers and cows. The heritabilities of NS and FSTC for heifers were higher than those for cows, while the heritabilities of GL and NRR for heifers were smaller than those for cows. These results were consistent with the results from a German Holstein population (Liu et al., 2008) but conflicted with results from a northern China population (Liu et al., 2017). Moreover, in previous studies, the genetic correlation between the NRR of heifers and the NRR of cows was almost 0.65 (Liu et al., 2017), which indicates that these traits should be treated as different traits, corresponding to genotype and environment interaction theories (Mulder, 2007). In the present study, the genetic correlation was 0.9993. One of the possible reasons for this result could be that the number of individuals used here was small for heifers. The other reason could be that the different populations’ genetic parameters were not consistent due to the procedure of selection, management, the climate of the location, and other residual errors. In a word, genetic parameter re-estimation was indeed necessary for different populations.

The heritabilities of the categorical traits (NRR, NS) were smaller than the interval traits, which was mainly caused by the linear model used for the variance components estimation for these traits. Though various non-linear models, such as the threshold model and generalized linear model, have been tested in the variance component estimation for NRR, ICF, etc., the previous results illustrated that the non-linear model might improve the heritability but decrease the reliability and increase computing time (Kadarmideen et al., 2000; Andersen-Ranberg et al., 2005; Sun and Su, 2010). The difference between the linear model and non-linear model could be negligible for multiple category phenotypes such as NS or non-extreme binary traits such as NRR in this study (Meijering and Gianola, 1985).

The benefits of a two-trait model for a certain trait depend on the heritability of this trait and the correlated trait and the genetic correlation between them (Schaeffer, 1984; Falconer and Mackay, 1996; Olasege et al., 2019). The genetic correlation of NRR for cows and heifers was 0.9993 and the heritability of NRR (cow) was relatively higher than the heritability of NRR (heifer). Therefore, the predictive ability for NRR (heifer) was expected to improve using the two-trait model in the prediction of estimated breeding values. When these two traits were merged into one trait, the heritability was 0.0026 ± .0.003. Therefore, the results suggested that we could merge the NRR from cows and heifers into one trait in the studied population.

It is well-known that a temperature–humidity index can be employed to assess and evaluate heat stress levels in livestock (Fathoni et al., 2022). Therefore, reproduction traits can be fitted in a reaction norm model to measure the impact of THI on reproduction traits. However, as the objective of this study was to estimate the genetic parameters of reproduction traits themselves, the THI was not considered.

CTFS and NRR56 have been suggested to be included in the selection index of the Chinese Holstein population according to the study from Liu et al. (Liu et al., 2017). However, as the heritabilities for these two traits were quite low in this study, it was hard to get genetic improvement when selecting these two traits. These differences could be because these two populations had different genetic backgrounds, and could be also because the size of records was small in this study. Since only a few individuals were overlapped for different traits, the two-trait model was not converged in this study. Therefore, genetic correlations were not obtained between traits. CI included the steps of CTFS, FSTC, and GL. Compared to the heritabilities of these traits, CI had higher heritability than CTFS and FSTC, which indicated that this complex trait in this population was more accurately recorded and had a high heritability. Considering the whether the heritability is high and whether the trait could be optional in a certain period, we suggest including NRR56, NS, AFC, and CI in the selection index in the current stage. Further study is necessary to collect more data to get genetic correlations and to update the selection index.

## Data Availability

The datasets presented in this article are not readily available because the data sets used in this study are the property of Shanghai BrightDairy & Food Co., Ltd. The data are available upon request with permission of the concerned institution through the corresponding author. Requests to access the datasets should be directed to PM, peipei.ma@sjtu.edu.cn.
